# Muscle force estimation from lower limb EMG signals using novel optimised machine learning techniques

**DOI:** 10.1007/s11517-021-02466-z

**Published:** 2022-01-14

**Authors:** Chiako Mokri, Mahdi Bamdad, Vahid Abolghasemi

**Affiliations:** 1 Corrective Exercise and Rehabilitation Laboratory, Faculty of Mechanical and Mechatronics Engineering, Shahrood University of Technology, Shahrood, Iran; 2 School of Computer Science and Electronic Engineering, University of Essex, Colchester, CO4 3SQ UK

**Keywords:** Rehabilitation robotics, Electromyography, Support vector machine, Support vector regression, Genetic algorithm, Random forest

## Abstract

The main objective of this work is to establish a framework for processing and evaluating the lower limb electromyography (EMG) signals ready to be fed to a rehabilitation robot. We design and build a knee rehabilitation robot that works with surface EMG (sEMG) signals. In our device, the muscle forces are estimated from sEMG signals using several machine learning techniques, i.e. support vector machine (SVM), support vector regression (SVR) and random forest (RF). In order to improve the estimation accuracy, we devise genetic algorithm (GA) for parameter optimisation and feature extraction within the proposed methods. At the same time, a load cell and a wearable inertial measurement unit (IMU) are mounted on the robot to measure the muscle force and knee joint angle, respectively. Various performance measures have been employed to assess the performance of the proposed system. Our extensive experiments and comparison with related works revealed a high estimation accuracy of 98.67% for lower limb muscles. The main advantage of the proposed techniques is high estimation accuracy leading to improved performance of the therapy while muscle models become especially sensitive to the tendon stiffness and the slack length.

**Figure:**
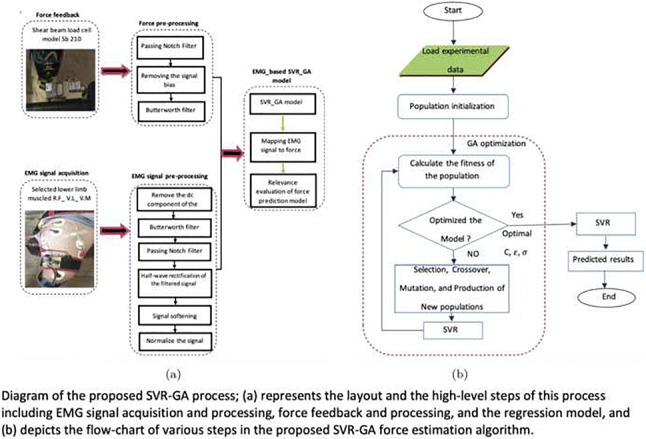
Graphical Abstract

Graphical Abstract

## Introduction

Rehabilitation is one of the most important types of cares that offers improvement or recovery of daily life abilities to patients [1, 2]. In particular, the use of robots in medical applications and rehabilitation in recent years has drastically increased. Vast amount of research is being conducted in this field for several purposes such as in upper [3] and lower [4] limbs of human body. Studies show that robots can significantly help physiotherapists in this area [5]. Physiotherapy is the physical or physiological treatment of the body that aims to improve the muscle function and stimuli. In physiotherapy, a therapist tries to restore the ability of the injured limb to perform normal movements by stimulating the neuromuscular and muscular stimuli. One of the disadvantages of this method is the difficulty imposed to physicians to carry out a repetitive errorless action without boredom and degradation in quality of service to the patient [6, 7]. Rehabilitation knowledge provides a useful framework for enabling the restoration of the motor abilities of patients with muscular weakness.

An accurate evaluation and successful treatment requires trained specialists as well as full rehabilitation facilities and equipment [8]. These activities then continue with assistive exercises where the patient and robot share the muscular force needed to move, and consequently, muscle strength increases.

Electromyography is the technique of obtaining electrical signals produced during muscle contractions. In fact, each of the muscles in the body is made up of a number of stimulating units that are responsible for contracting the muscles and producing strength in the muscles. Muscle contraction can be detected during neural activity by recording and analysing EMG signals [9]. Surface EMG reflects the amount of musculoskeletal electrical activity in a noninvasive manner. This signal is highly correlated with muscle strength under certain circumstances and motor tasks. Therefore, it could be used as a means of measuring bioelectrical events associated with muscle fibre contraction. For example, in prosthetic control, a more accurate imitation of the natural command is expected to be provided between the central nervous system and the motor system [10]. Assistive robotic devices use these signals as control inputs [11].

Estimation of muscular force is one of the ongoing research topics in the field of biomechanics with the aim of enhancing clinical decision making [4, 12, 13]. In force-based physiotherapy, feedback should always be taken from the muscular force to have a system with minimum possible error [14]. The integration of various sensors’ data and the evaluation of the patient’s clinical reactions with the help of active and passive rehabilitation robots has been studied in recent years [15]. As a result, these systems must be user-centered and controlled by taking into account the user’s dynamic specifications, and EMG signals [16]. In fact, EMG signals can provide information about the difficulty of the associated task for the user. Hence, if the muscular force can be estimated with least possible error, the accuracy of applying force to the user’s limb via the prosthesis will be increased. In this regard, Kigochi et al. provided an EMG-based approach to control the upper limb body robot in accordance with the user’s intent to move and relocate [17]. They attempt to take into account both characteristics of EMG signals and human body in their method. Deep learning techniques have been also used for this propose [18, 19]. For instance, a deep neural network is proposed in [20] to learn mapping from movement space to muscle space. In [19], an estimation model for upper limb joint angle based on deep learning is proposed. The authors use sEMG and studied the touching motion and the compound task motion of the upper limbs. The main challenge with deep learning methods is that large amount of training data is needed which is not always the case.

Surface EMG is affected by physiological factors such as phase neutralisation and improper placement of electrodes. These factors subsequently affect the accuracy of force estimation by the sEMG [21]. External factors such as motion artifacts, ambient noise, and electrical equipment noise can also affect the quality of the acquired sEMG signals [22]. Therefore, it is very important to devise suitable pre-processing methods to mitigate the defects of such noises. Various techniques have been proposed for this purpose. The pre-processing techniques commonly involve some filtering, full-wave rectification and wave smoothing [23, 24]. The main task after pre-processing stage is to model the EMG signal(s) into the desired output. This can be normally divided in two forms of *classification* or *estimation*. In classification, the input signal is normally categorised into several classes depending on the tasks performed by subjects during the signal acquisition. On the other hand, estimation techniques seek for ways to predict the muscle force, exerted by the associated limb, from the EMG signals. Different modeling techniques have been used to accurately estimate muscle strength using EMG signals. The existing models can be categorised in different ways. For example, there are some input-output models (black box models), including artificial neural network (ANN) and support vector machine [25–27]. One of the effective factors in creating muscle strength in various functions is the speed of muscle contraction. The relationship between isotonic power and the rate of muscle contraction has been introduced by Fan and March through an exponential model [28]. Another class of models is called physic-based model such as Hill-type model, which has been proposed to predict the force created in muscle fibres at different speeds [29]. In this model, the assumption is that the estimated muscle force is proportional to the muscle activation, therefore, the transformation from EMG to muscle activation is modeled. There are also some studies on the longitudinal effect of muscle fibres on muscle strength. Among these studies, the works conducted by Edman et al. [30] and Deleuze [31] can be mentioned. Expansion of these studies has led to the presentation of various mathematical models for predicting muscle strength such as multi-component relationships [32, 33]. Hashemi et al. used the parallel cascade detection (PCI) modeling method to estimate the muscular force induced in the wrist based on the EMG signals of the arm muscles [34]. Shabani and Mahjoub were able to improve the capabilities of the designed robot by using the EMG signals obtained from the knee [35].

In recent experimental studies, the researchers use knee rehabilitation devices to further exploiting the patient’s neuromuscular abilities and predict muscle strength and active torque of the knee joint [35]. In addition, quadriceps femoris strength issues are common in individuals with knee joint impairments after injury or surgery [36]. Therefore, estimating the torque of the knee joint muscles by the neural network and various methods is one of the most important steps to improve the control function of human interaction in the knee rehabilitation robot [37]. Using ANN, Khanjani et al. were able to estimate lower limb forces using EMG signal [38]. In another study, the authors were able to calculate the amount of knee torque by a support vector regression based on electromagnetic signals [39]. Wang et al. were able to calculate the parameters of the backup vector regression method using particle density (PSO) optimisation method [40]. Meng et al. also used the SVR model to predict the lower limb force by EMG to control the rehabilitation assistant robot [41]. The main issue in using existing SVR-based techniques is appropriate selection of model parameters which is normally performed manually.

In this paper, the aim is to estimate the exerted force by knee muscles from acquired sEMG signals. We address both classification and estimation problems by applying appropriate machine learning techniques to the data obtained from our designed rehabilitation device. For classification task we apply techniques based on support vector machine. For estimation, we propose using support vector regression and random forest. Moreover, we propose using genetic algorithm for improving the features extraction and parameter optimisation in the aforementioned methods. The contributions of this work are listed below:

- Designing and implementing an experimental set up to simultaneously record sEMG and force signal of knee movement at any arbitrary angle.
- Data recording of several subjects with the designed protocol.
- Classification of EMG signals and estimation of muscle force signal from sEMG signals.
- Utilising three main machine learning techniques, namely, SVM, SVR and RF.
- Using Genetic Algorithm to obtain suitable features from the input signals to improve the classification accuracy.
- Using Genetic Algorithm to optimise and tune the parameters of both classification and prediction models.

The rest of the paper is organised as follows. Section 2 represents the system design and implementation details, including the rehabilitation device specifications and data acquisition process. In Section 3, the proposed machine learning techniques used in this research will be described. In Section 4, our proposed approach for optimising the model parameters using genetic algorithm will be discussed. Section 5 is devoted to the experimental results for both classification and prediction. Finally, a conclusion is drawn in Section 6.

## System design and implementation

### Knee rehabilitation device

**Fig. 1:**
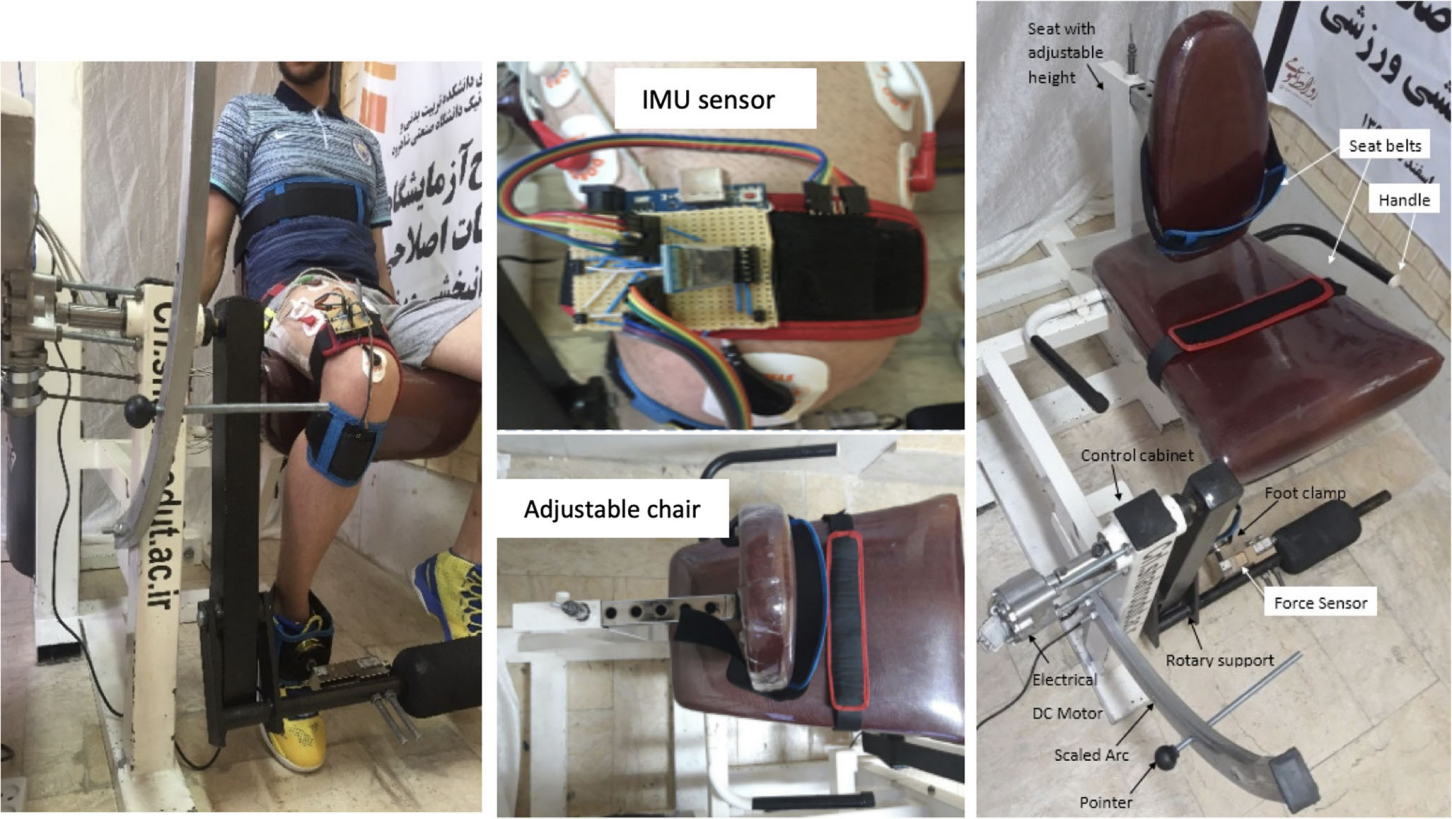
The architecture of knee rehabilitation device with annotated details

Single-joint training is chosen when improvement of functional ability of a specific joint is required. The knee rehabilitation device is a robot of one degree of freedom that is designed and implemented to rehabilitate the knee joint and leg muscles (Fig. 1). The robot consists of mechanical, electrical, control, and medical equipment. The mechanical part includes an adjustable seat, belt, force measurement mechanism and calibrated arc with angles of 30, 60, and 90^∘^. Single-joint training is usually selected for range of motion exercise. These angles were calculated by the designed inertial measurement unit (IMU) located on the user’s knee as an electrogoniometer. The control section also includes the electric motor, the driver and the interface board. Medical equipment includes EMG recording devices, surface electrodes, conductive gels, and so on. A computer connected to an EMG device and an Arduino board were used to receive force data from a load cell (shear beam model-SEWHA-SB210) followed by an A/D converter (HX711). In order to perform physiotherapy exercises and knee rehabilitation, the patient sits on a chair and place their foot on the load cell fixed by the brace. The patient-centered orientation of the patient with the rotational axis of the robot is very important in the correct measurement of the patient’s knee angle. During training, sEMG signals and the corresponding force signals are simultaneously recorded.

Isokinetic dynamometer as a testing method that requires extensive training is reliable in assessing muscle strength and the estimated joint torque is calculated from the sum of the partial torque contributions provided by the muscles. In comparison, this relatively inexpensive measurement system is used in clinical practice through hand held dynamometers for the examination of muscle testing. It has been established as a reliable and cost-effective measurement technique in experienced hands under controlled conditions. Moreover, it shows more effective result rather torque calculation for muscle force estimation and provides a means of adjustability and sensor portability for clinicians. To appropriately measure muscle strength, the sensor type, shape and sensitivity are determined depending on the type and shape of the target limb and the direction of its movement and the amount of force applied by the limb. The location of the sensor also plays an important role in accurately measuring muscle strength [42].

### Data recording

Five healthy individuals with no history of illness, diet, or medication were invited to take part in the experiments. The age and height characteristics of the individuals were measured, and their mean and standard deviation were 23.5 ± 4.5 years, and 174.10 ± 4.3cm, respectively. In this experiment, 60^∘^ isocentric knee exercise performed on 5 healthy people. All volunteers were subjected to sEMG analysis and examined in universities’ corrective exercise and rehabilitation laboratory (CRL) according to the approved protocol. The testers were also directly supervised by a clinical faculty mentor. The subjects where asked to first wax their right leg and then sit on a designed knee rehabilitation device and place their right foot on the force sensor. The leg is fully fixed by the brace. During the exercise, the person was asked to apply force to the load cell for 5 s to relax and open the knee and rest for 5 s. These steps were performed 3 times in a row (without fatigue) and sEMG signals of the quadriceps femoris muscle contraction and its corresponding force were recorded.

**Fig. 2:**
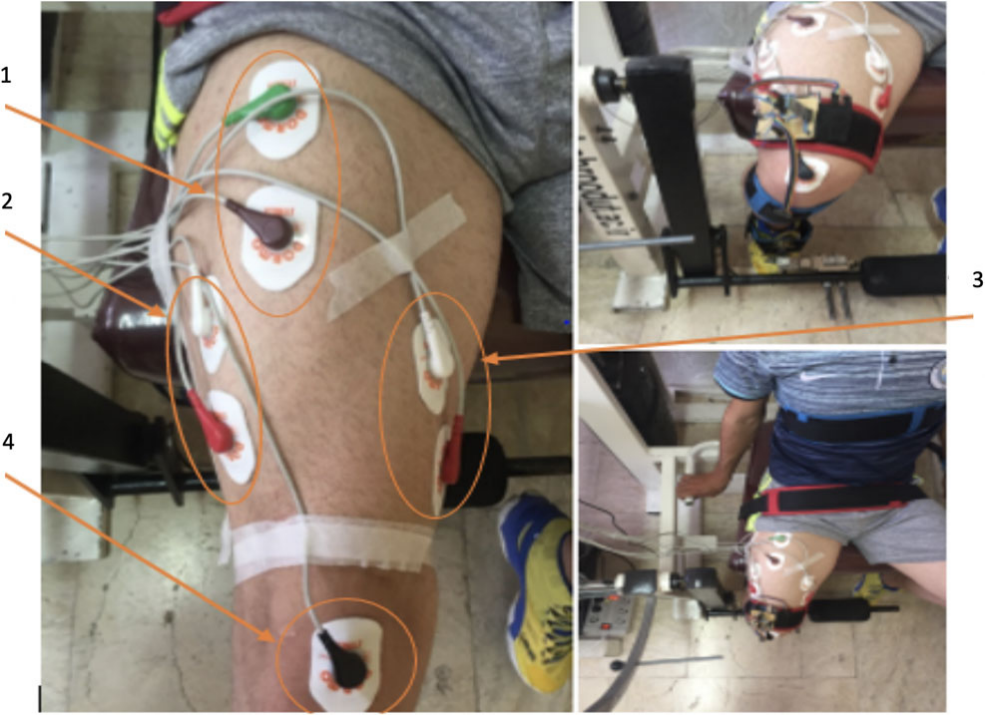
Electrode locations and detail: (1) rectus femoris; (2) vastus medialis; (3) vastus lateralis; (4) zero reference electrode

To record the sEMG signals, an 8-channel EMG signal recording device is used. Ag-AgCl surface electrodes were also used. sEMG signals, according to the SENIAM standard,^1^ are recorded from three quadriceps muscles: vastus medialis (VM), vastus lateralis (VL) and rectus femuris (RF) with the sampling frequency of 1 kHz (Fig. 2). The lower-limb muscles of invited subjects involved in the knee exercise are checked and the exact locations of the electrodes are identified. Due to the wide frequency range of the EMG signal variations, skin cleansing is essential to reduce sensing impedance. For this purpose, the skin surface must be cleaned of dead cells with a soft sandpaper before installing the cast leads. The skin was then thoroughly cleansed by alcohol. Calibration of the load cell was performed using a pressure device where a continuous force in the range 1-to-300 *N* is exerted to the load cell over time. At the same time, the load cell output was being collected at every second. This gives proportion of the exerted force against the movement at a constant velocity which is considered as a baseline. Then, the force exerted by the subjects in the real experiments and the movement of the knee are recorded and compared with the baseline.

It is desirable that the recorded EMG data and the measured force by the load cell be used respectively as input and output of the proposed models. The aim of this setting is to compare the measured source with the proposed sEMG and analyse the performance and accuracy of the system.

## Materials and methods

In this study, we propose SVM, RF and their variants as classifiers for finding the correct data class, i.e. active and rest. Further, SVR and optimised SVR using genetic algorithm are proposed for estimation of the force signal from the recorded sEMGs. In this section, we first give details of the pre-processing steps applied to both EMG and force signals. Then, three different supervised classification techniques, i.e. support vector machine, support vector regression, and random forest, will be explained.

### Pre-processing module

We follow common state-of-the-art pre-processing techniques in order to remove unwanted noise and artifacts from both EMG and force signals [43, 44]. Typically, low frequencies 1-20Hz which do not involve important information and are corrupted by movement artifacts should be rejected. Radiation from power sources, which is also called Power-Line Interference, is an ambient noise arising at 50 or 60 Hz. The impact of this noise can be mitigated by applying a narrow-band notch filter. Overall, these steps which help to eliminate noise and prepare the EMG signal for force estimation are applied in our study:

1. Removing the DC component of the signals.
2. Band-pass butterworth filtering with cut off frequency of 20 and 500 Hz.
3. Passing the narrowband notch filter.
4. Half-wave rectification of the filtered signal.
5. Signal softening.
6. Signal normalisation.

The pre-processing steps for the force signal are as follows:

1. Passing notch filter.
2. Removing the signal bias.
3. Softening with a two-level low-pass butterworth filter with cut off frequency of 15 Hz.

**Fig. 3:**
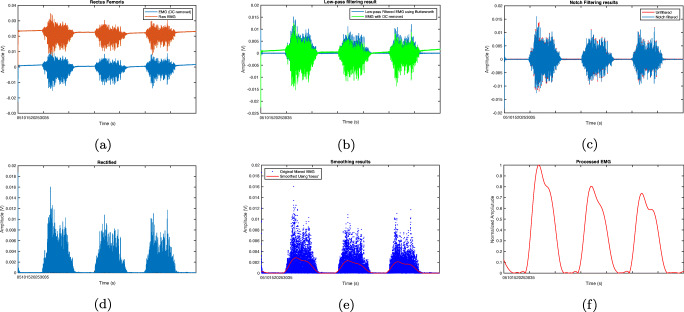
EMG pre-processing example for subject 1 (S1) and rectus femoris: (a) DC removal, (b) butterworth filtering, (c) notch filtering, (d) rectification, (e) smoothing, (f) normalisation

**Fig. 4:**
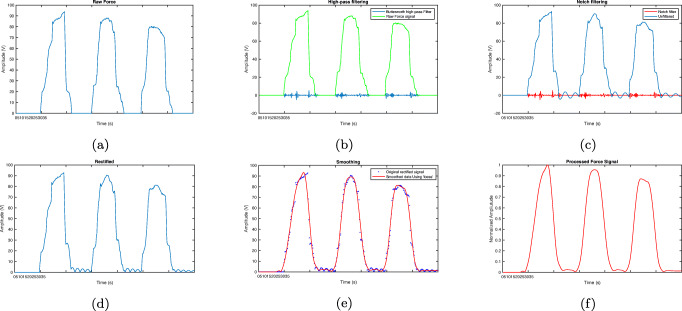
Force signal pre-processing: (a) raw signal, (b) butterworth filtering, (c) notch filtering, (d) rectification, (e) smoothing, (f) normalisation

In order to clearly observe the effects of each pre-processing step, Figs. 3 and 4 are given as samples of sEMG and force signals, respectively. As seen from Fig. 3(a), the raw sEMG signals are noisy but when filtered through a two-stage process, the extreme frequency contents are removed. The rectification and smoothing stages make the signal ready for feature extraction Fig. 3(f). The same sequence of pre-processing steps are applied to force signals (Fig. 4). This figure shows that raw signal include some noise and fluctuations but the pre-processed signal is smooth and ready for further processing (Fig. 4(f)). In both figures, the pre-processed signal (i.e. Figs. 3 and 4 (f)) are used as inputs of our machine learning models which will be described next.

### Classification module

In the following subsections, we explain the working mechanisms of the classifications techniques we applied to the pre-processed sEMG data. First, support vector machine will be briefly described followed by random forest technique.

#### Support vector machine

Support vector machine is a supervised learning method used for classification and regression. It was first introduced in 1992 by Vapnik and his colleagues based on statistical learning theory [45]. The basis of SVM classification is linear data separation where samples of the different classes are separated by a hyper plane. The use of support vector machine is mainly in cases where the data is not linearly separable in their current domain. SVM transforms the input data points to a feature space where can be linearly separated. In fact, it separates the classes by introducing support vectors to maximise the distance between the samples of different classes. Thus, it is also referred to as large-margin classification. In general, there exist several hyper-planes that can separate the data samples. What makes the support vector machine different from other classifiers is how it selects the hyper-plane. In a support vector machine, the objective is to find the maximum margin between the two classes. Therefore, it selects a hyper-plane in which distance from the nearest data on both sides of the line separator is maximised. If such a hyper-plane is identified, it is called maximum-margin hyper-plane. The decision-making function for separating data is determined by a subset of closest training data to the hyper-plane, called support vectors. In fact, the optimal hyper-plane in a support vector machine is a separator between support vectors. Due to simplicity and flexibility of SVM we are interested to apply it for sEMG classification. However, we propose an intelligent method, based on genetic algorithm, to automatically select and tune appropriate features and parameters, respectively.

#### Random forest

##### Definition

**Fig. 5:**
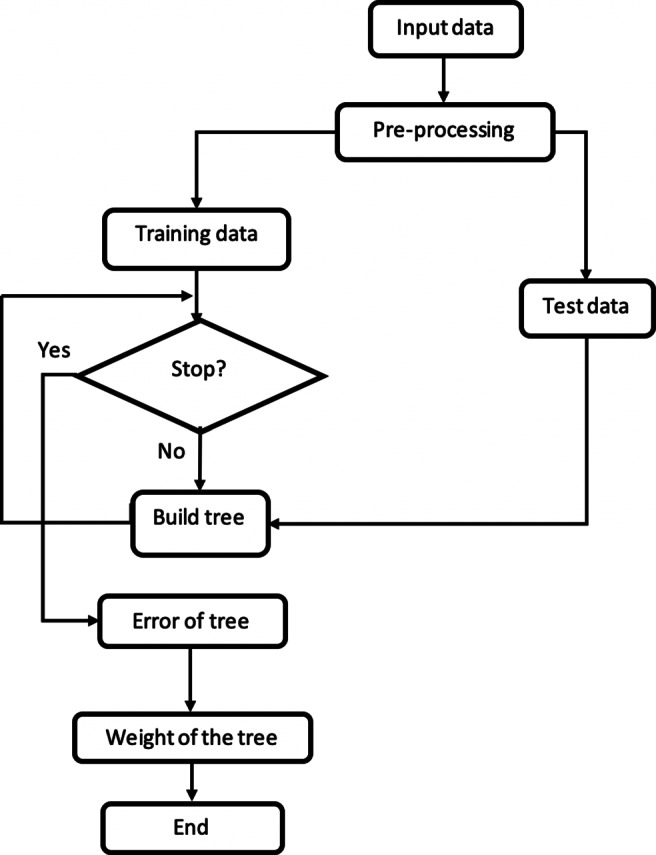
Flow chart of random forest algorithm

Random forest method is categorised as a supervised ensemble learning technique [46]. Ensemble learning refers to the process by which multiple models, . experts, classifiers, etc., are combined and work together to solve a particular computational intelligence problem. Hence, RF produces several different decision trees as basic classifications and applies the majority vote to combine with the results of the main trees [46]. Random forest works based on multiple deep decision trees as classifiers. Each classifier associated to an input sample is shown by *h*(*x*, *θ*_*k*_), where *x* is an input sample and *θ*_*k*_ is the training set for the *k* th tree. The *θ*’s are independent of each other but with the same probabilistic distribution. For each sample *x*, the corresponding tree provides a class prediction, and finally the class with the highest number of tree votes on input *x* is selected as the winning class. The flow chart of RF algorithm used in this work is shown in Fig. 5. The random forest algorithm increases the accuracy of the individual classification tree. In an individual tree, small changes in the training set generates some instability, which disrupts the accuracy of the prediction in the experimental sample. But group of random trees (random forest) adapts to change and eliminates instability.

##### Out of bag estimation

Out of bag (OOB) estimation is a method to evaluate the prediction error of random forests. It utilises bootstrap aggregating to sub-samples of data that used for training. Suppose each classifier with a new training set is built using a decision tree method. The training sub-sets *θ*_*k*_ are formed according to the main training set *θ* using bootstrap technique. Then, the *h*(*x*, *θ*_*k*_) tree classifiers are created, and each tree is voted for predicting the right class. Those training samples which do not belong to the k-class training set are called the out-of-bag (OOB) samples. Equation 1 shows the estimate approach for the OOB samples of the forest. In order to obtain the sample class, the prediction of the trees whose training set does not contain the sample must first be recognised, and then the category with the highest average vote on the forest tree predictions will be considered as the corresponding sample class.

1 $$ y(x)=\arg\max_{c}\left( \frac{1}{k}\sum\limits_{k=1}^{K} I(h_{k}(x)=c,x\in OOB_{k})\right) $$

2 $$ I(h_{k}(x))\!=c, \ x\!\in\! OOB_{k} = \left\{\begin{array}{ll} 1, & \text{if $h_{k}(x)=c, x\in OOB_{k}$}.\\ 0, & \text{otherwise}. \end{array}\right. $$

where *k* is the number of trees, *c* is the class index, *h*_*k*_(*x*) is the prediction of the *k* th tree from the sample *x*, and *OOB*_*K*_ is the set of OOB for *k* th tree samples. Equation 2 shows that the value of the index *I* function will be one if *x* is among the samples of the *k* th tree (i.e. not a member of the *k* th tree training set). Also the *k* th tree classifies the sample *x* into class *c*. Otherwise, the value of the index function *I* becomes zero. To estimate the OOB samples on the forest, we first modify Eq. (2) into Eq. (3), and then use *ε*_*k*_ in Eq. (4), which is a classif ication error of the forest on the OOB samples of the *k* th tree. *N* is the total number of samples of the main training and *x*_*i*_ is the *i* th sample on the main training set.

3 $$ I((y_{i},x_{i})\in OOB_{k})=\left\{\begin{array}{ll} 1, & \text{if $(y_{i},x_{i})\in OOB_{k}$}.\\ 0, & \text{otherwise}. \end{array}\right. $$

4 $$ \varepsilon_{k}(OOB)=\frac{{\sum}_{i=1}^{N}(y(x_{i})=y_{i},(y_{i},x_{i})OOB_{k})}{{\sum}_{i=1}^{N}I((x_{i},y_{i})\in OOB_{k}} $$

### Estimation module

Although SVM and RF can be used for classification of the state of the subject, they cannot be used for evaluating the system performance based on the measured muscle force. Support vector regression (SVR) can be used in this regard as it solves predictive and estimation problems [47]. In order to use the support vector machine in regression problems, Vapnik used an loss function that ignores errors at a certain distance from the actual values denoted by *ε* = *y* − *f*(*x*, *α*) [45], where *x* is the input data and *f*(*x*, *α*) is the response (a set of indicator functions). This loss function is then defined as follows:

5 $$ L(y,f(x,\alpha))=\left\{\begin{array}{ll} 0, & \text{if $y=f(x,\alpha)$}.\\ 1, & \text{if $y\neq f(x,\alpha)$}. \end{array}\right. $$

Now, let us consider the approximation problem for the following set as:

6 $$ D=\{(x^{1},y^{1}),{\cdots} (x^{t}, y^{t})\}, \ \ x\in\mathbb{R}^{n}, y\in\mathbb{R} $$

The regression function is estimated as follows:

7 $$ f(x) \sim <w,x>+b $$

where <> denotes the inner product between two vectors (*w* and *x* are the weight and input space vectors, respectively). The optimal regression function can be defined by minimising the following function:

8 $$ \phi(w,\alpha)=\frac{1}{2}\|w\|^{2}+C{\Sigma}_{i}(\xi_{i}^{-}+\xi_{i}^{+}) \ s.t. \ \left\{\begin{array}{l} y_{i}-(<w,x_{i}>+b)\leq \varepsilon+\xi^{+}_{i}\\ \xi^{+}, \xi^{-}\geq 0 \end{array}\right. $$

where *C* is a predetermined value, and $\xi _{i}^{-}$ and $\xi _{i}^{+}$ are slack variables that determine the upper and lower constraints of the system output. If the data is represented as separate outputs, it provides an optimal level that separates the data without error and with the maximum distance between the hyperplane and the nearest training points (support vectors). If we define the training points as *x*_*i*_ and *y*_*i*_, and the input vector as $x_{i}\in \mathbb {R}^{n}$, where the data is linearly separable, the equation would be as follows:

9 $$ y=f(x)=sign({\Sigma}_{i} \alpha_{i} <w,x_{i}>+b) $$

where *y* is the output and *y*_*i*_ is the value of the associated class to training sample *x*_*i*_. The vector **x** = [*x*_*i*_, *x*_2_,⋯*x*_*n*_] represents an input vector. The values *x*_*i*_;*i* = 1,2,⋯*N* are support vectors. If the data is not linearly separable, it would be possible to transform them to a higher space by applying some pre-processing. In this case, the equation (9) is converted to:

10 $$ y=f(x)=sign({\Sigma}_{i} \alpha_{i} k(x,x_{i})+b) $$

The *k*(*x*_*i*_, *x*), is a kernel function that generates several inner products to create machines with different types of nonlinear surfaces in the data space. Various kernels are used for the regression model of the support vector machine, which are: linear, polynomial and radius basis function (RBF) kernels. Normally, the Gaussian radius basis function is more appropriate for predicting [45]. The equation for this kernel is as follows:

11 $$ k(x,y)=\exp{\frac{||x_{i}-x||^{2}}{2\sigma^{2}}} $$

## Proposed model selection and parameter tuning

In the previous section, the techniques employed in this study for EMG *classification* (i.e. SVM and RF) and force *estimation* (i.e. SVR) were described. However, there are two major challenges in maximising the performance of these methods: (i) selecting the most appropriate features, and (ii) optimising the tunable parameters. This section is devoted to provide suitable approaches for model selection and parameter optimisation. We propose to use genetic algorithm for two important tasks, i.e. feature extraction and parameter optimisation. The procedure of the proposed approach is as follows. The support vector machine model is formed using the training data and the calculated parameters (by GA) for desired hyperplanes. Then, in order to calculate the objective function, the test data is classified by the trained SVM model and an error matrix is formed. After evaluating the members in GA, three steps of selection, integration and mutation are performed on the binary format of the parameters and a new population is created and these steps are repeated to establish the condition of stopping.

### Genetic algorithm

Genetic algorithm is a powerful method to solve problems for search and optimisation. GA attempts to simulate evolutionary behaviours of nature. This algorithm works with a population of unique members, which defines a fitness value for each member. Obviously, members with higher fitness are more likely to be engaged with others and generate new members. The created new members inherit certain characteristics of their parents. Also, the less fitness of a member of the population is, the less likely it is to be selected for reproduction. By selecting the best members from the current population and merging them, a new set of members is created, which has a relatively higher rate than the previous population. As this process continues, after several reproductions and consecutive populations, the members’ attributes are gradually disseminated in the populations, and the members are optimally modified. So far, numerous GAs have been successfully applied to solve a wide range of problems. Of course, these algorithms do not guarantee a general optimal solution to all problems, but they always act as a strong tool to find solutions that are reasonably acceptable [48]. The basic principles of a genetic algorithm include:

1. Production of primary population including n chromosomes;
2. Investigating the evaluation function *f*(*x*) for each chromosome *x* in the population;
3. Creating a new population based on the repetition of the following steps:
  Select two parent chromosomes from a population based on their suitability.
  Consider a certain amount for the crossover probability and then perform the recombination on the parents in order to create children.
  Consider the possibility of mutations and then change the children in each place.
  Replace new children in the new population.
4. Using a new population for the next runs of the algorithm;
5. Stopping the execution of the algorithm if the stopping conditions are met and returning the best solution in the current population, otherwise going to step 2;

### Feature extraction in SVM/SVR based on GA

Feature selection is one of the most effective steps in classification based on support vector machine, in which eliminating irrelevant bands improves class performance in terms of accuracy and speed. In this section, we apply feature selection from sEMG signals using the genetic algorithm. This is examined in the presence of all input sEMG signals. Then, the main process of selecting the optimal features is determined by binary coding of the parameters. In order to define a criterion for evaluating the quality of a subset of selected features, two parameters of classification accuracy and the number of selected features must be considered. In other words, a desired classification would include the subset of the most effective attributes as well as lower number of selected features. Therefore, we propose an objective function by representing these two criteria in a closed form which is to be maximised (Eq. (12)). In fact, in this method, we are going to use the genetic algorithm to see what features are more important and significantly improve the classification accuracy.

12 $$ f=W\times Acc+(1-W)\times \frac{1}{N_{f}} $$

In the above equation, *f* is the objective function, *W* is the weight in the range of [0,1] which controls the contribution between the number of features and classification accuracy, and *Acc* represents the accuracy of the classification. After evaluating the population, the three selection, merging, and mutating operators act according to the quality of each member, and again the population created by the objective function will be evaluated, and this process is repeated to establish a stopping condition.

### Parameter tuning using genetic algorithm

Optimal selection of parameters in a SVM/SVR model has significant impact on the overall performance of the method. There are two sets of parameters to be identified in our model: (i) adjustment parameters, that mainly balance the error and complexity minimisation of the model (. C), and (ii) kernel parameters, which are unknown and have to be found (. *σ* which is the most important parameter in Gaussian kernel (Eq. (11)).

Support vector machines are intrinsically binary classifiers. From this perspective, the existing algorithms for determining the parameters of SVM, the so-called model selection, are also divided into two categories. In the first category, the same set of parameters is considered for both classes. While in the second category, different parameters are determined for each binary class. Adding unknown parameters, in most cases, not only decrease the accuracy of the classification, but limit the classification performance due to overfitting the model. In recent years, various methods have been proposed to determine the optimal parameters in SVMs. Network search algorithm is a common method for selecting the optimal model. Due to the continuity of the values of the desired parameters, a high-density network must be considered to achieve a high accuracy. This is to examine all these points of the network which greatly increases the computation time. Due to these limitations, other tuning algorithms have been generally considered to solve related problems: collective micro-algorithms, simulation of gradual refrigeration, and genetic algorithms. Genetic algorithms are meta-heuristic techniques that have been used successfully and extensively to select the optimal model parameters in support vector machines [49].

**Fig. 6:**
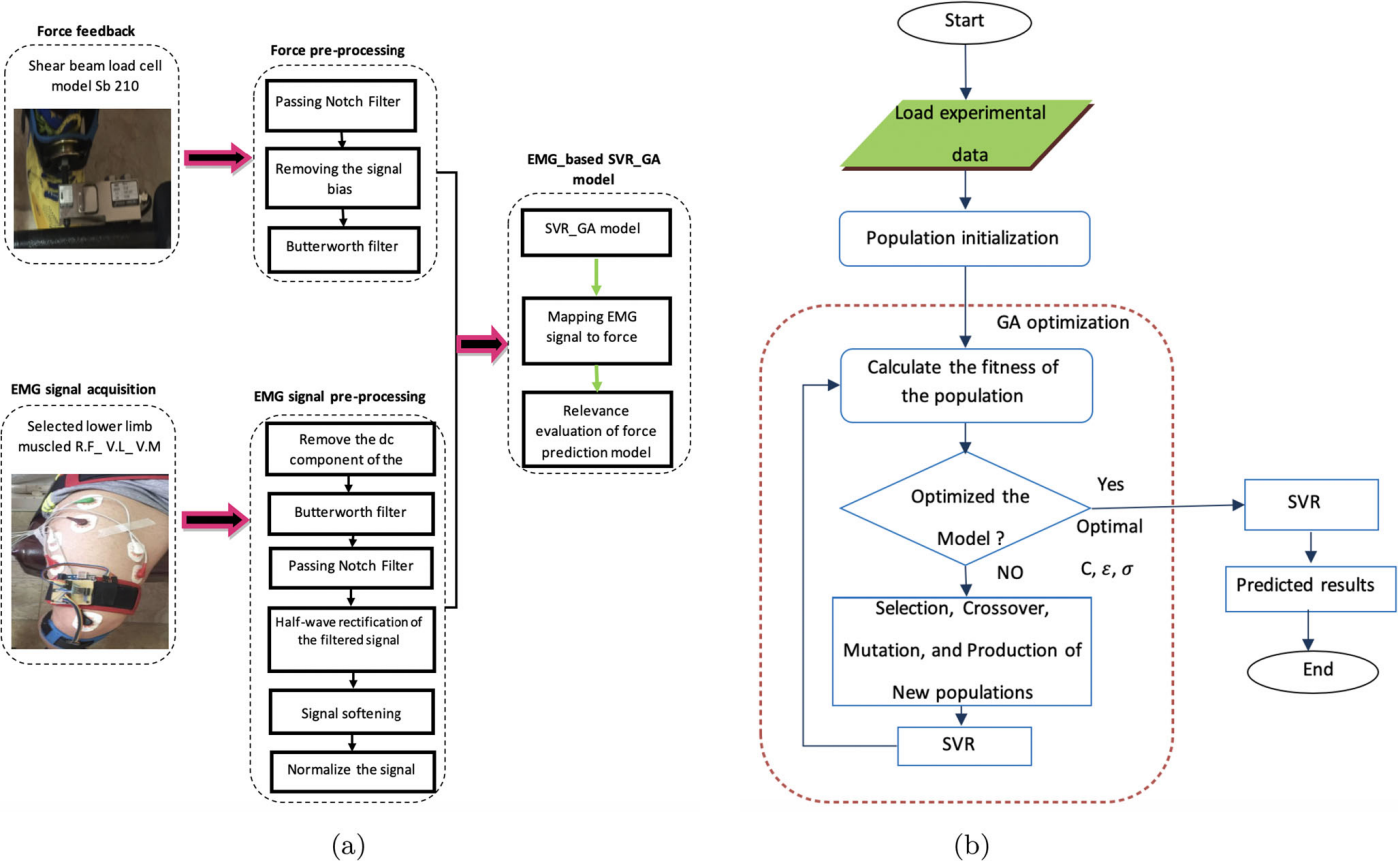
Diagram of the proposed SVR-GA process; (a) represents the layout and the high-level steps of this process including EMG signal acquisition and processing, force feedback and processing, and the regression model, and (b) depicts the flow-chart of various steps in the proposed SVR-GA force estimation algorithm

In this paper, support vector machine and regression as well as random forest models have been used for classification and to force prediction from surface EMG signals. In such intelligent modeling systems, classification/prediction accuracy is largely dependent on model learning parameters; therefore, the genetic algorithm has been used to find the optimal parameters in this model. In order to properly evaluate the quality of any member in GA, we convert both parts of the chromosome to a real number. In the next step, the support vectors for both SVM and SVR will be formed by using the training data as well as the tuned parameters of the corresponding hyper-planes. Then, the test data is given to the obtained model in the training step and an error matrix is formed. After evaluating the samples, three steps of selection, integration and mutation are performed on the binary format of the parameters and a new population is created. These steps are repeated to establish the stopping condition. In this way, the parameters *C*, *ε* and *σ*^2^ are optimised. Full details of different steps involved in our proposed SVR-GA are depicted in Fig. 6.

## Experimental results

The experiments have been conducted according to the procedures explained in Section 2. Through all experiments and the entire processings, we have used 3-channel EMGs from quadriceps muscles, namely VM, VL and RF, per each subject. The exemplar graphs and signals illustrated in this section are associated to RF muscles and we omit plotting the signals of VM and VL to avoid duplication. In the sequel, we report the obtained results in two different classification and estimation modes. The numerical results within the tables are given for all the subjects. However, signal illustration of specific subjects and lower-limb muscles is provided where appropriate.

### Classification performance

**Fig. 7:**
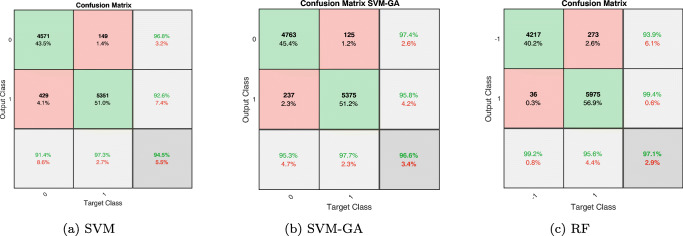
Confusion matrix result for S1 with various classification methods. (a) SVM. (b) SVM-GA. (c) RF

In the first experiment, we aim to assess the performance of SVM and its variants. We empirically select *C* = 1 and *α* = 0.5 with Gaussian kernel for this classifier. Then, the pre-processed sEMG signals are given to the SVM model. The accuracy of this two-class classification with data from all five subjects is given in Table 1. According to this table, the classification accuracy of the proposed method for all subjects are promising and average classification accuracy of 93.14*%* has been achieved. In this experiment, we have used 70*%* and 30*%* of force and EMG data for training and testing phases, respectively. A 10-fold cross validation procedure has also applied to ensure a reliable model verification. In Fig. 7(a), the confusion matrix of the classifier’s output (for subject S1) with respect to the target class is illustrated. As seen from this figure, the average classification accuracy is given in the last row and column. Values in the green boxes show the percentage of the associated class data with respect to the total data. For example, it can be seen from Fig. 7(a) that out of 4568 samples in class 0 (equivalent to 43.5*%* of total data) 91.4*%* has predicted correctly. Also, out of 432 samples in the same class (equivalent to 4.1*%* of total data), 8.6*%* has predicted incorrectly. The same interpretation can be made for the results of class 1 in Fig. 7(a).

**Table 1:** Classification results of SVM for all five subjects

Subject	S1	S2	S3	S4	S5
Accuracy(%)	94.5	93.1	93.0	92.7	92.4

In the next experiment, we aim to observe the effects of using optimised SVM parameters by GA instead of manual parameter selection. Hence, we apply the proposed SVM-GA algorithm under similar conditions as previous experiment to the data from the five subjects. In the proposed genetic algorithm, main population was chosen as 20 and the iteration of the target function was selected as 50. Figure 7(b) shows the confusion matrix associated to the SVM-GA results with *C* = 2 and *α* = 1.5 (obtained by GA) on S1. It can be seen that the average accuracy is 96.6*%* which is higher than that obtained in the previous experiment (Fig. 7 (a)). The classification accuracy of all subjects in addition to the obtained parameters from GA is given in Table 2. Comparing the results of Tables 1 and 2 confirms the superiority of the method when the parameters are obtained using GA.

**Table 2:** Classification results of SVM-GA

Subjects	S1	S2	S3	S4	S5
Accuracy (%)	96.6	95.0	94.6	93.2	92.9
Optimised C	2.00	1.86	2.21	1.43	1.78
Optimised *α*	1.51	1.22	2.50	1.00	1.90

**Fig. 8:**
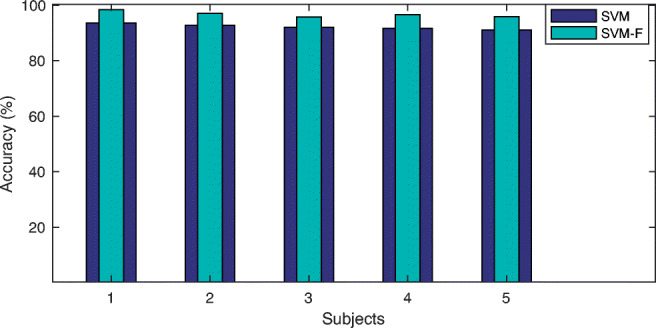
Comparison of classification accuracy when direct samples (SVM) and selected features (SVM-F) are used

In this paper, GA was used for both parameter optimisation and also feature selection in SVM. In order to explore how GA can affect the classification performance by appropriate feature selection in SVM another experiment was conducted. Similar to previous experiment, here we have used GA with 20 initial population and 50 number of iterations. Also, we used 70*%* of samples for training and 30*%* for testing phases. The results of two cases are presented in Fig. 8: (i) data samples with all features are used for classification (SVM), and (ii) selected features via GA is used for classification (SVM-F). As observed from Fig. 8, the classification accuracy is increased when features selected by GA is used for classification using SVM.

**Fig. 9:**
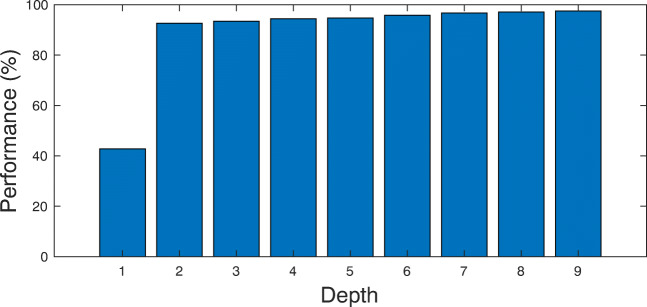
RF classification accuracy against various Depths

In this part of experiments, we evaluate the performance of random forest (RF) for classification of EMG signals. The best results were achieved by empirically selecting *K* = 50 and Depth = 9 in this algorithm. The corresponding results are presented in Table 3, where as seen, high accuracy has been achieved. The average accuracy among all subjects is 95.26*%*. In order to illustrate the robustness of RF against different selection of depth parameter, we demonstrate the classification accuracy for different depth values in Fig. 9. It can be seen that for Depth > 7 no significant improvement is achieved. The confusion matrix for S1 classification in this experiment is shown in Fig. 7(c) where 97.1*%* accuracy has obtained. According to Fig. 7, the proposed RF classification outperforms both SVM and SVM-GA.

**Table 3:** Classification accuracy using RF method

Subjects	S1	S2	S3	S4	S5
Accuracy (%)	97.1	96.4	95.0	94.3	93.5

Finally, we provide a table that compares average performance across all subjects for different techniques we have applied so far, i.e. SVM with no feature selection (SVM), SVM with optimised parameters (SVM-GA), SVM with optimised parameters and selected features (SVM-GA-F), and random forest (RF). As seen from Table 4, applying SVM with selected features and optimised parameters using GA provides the best performance.

**Table 4:** Comparison of average classification accuracy for different methods

Method	SVM	SVM-GA	SVM-GA-F	RF
Accuracy (%)	93.4	94.4	96.7	95.2

### Estimation performance

In this section, we aim at evaluating the estimation performance of the proposed system.

It is noted that we input the pre-processed sEMG signal into the proposed estimation model (based on SVR and SVR-GA), and then the output of this process will be compared with the corresponding measured force signal (Fig. 6). The performance measures we have considered to demonstrate the influence of each step on the final force estimation process are as follows:

- Mean-Square-Error (MSE):
  13 $$ \text{MSE}=1/n\sum\limits_{i=1}^{n}(\hat{y}_{i}-y_{i})^{2} $$
- Root-Mean-Square-Error (RMSE):
  14 $$ \text{RMSE}=\sqrt{MSE} $$
- Mean-Absolute-Error (MAE):
  15 $$ \text{MAE}=1/n\sum\limits_{i=1}^{n}|\hat{y}_{i}-y_{i}| $$
- Relative-Standard-Error (RSE):
  16 $$ \text{RSE}=\sqrt{\frac{{\sum}_{i=1}^{n}(\hat{y}_{i}-y_{i})^{2}}{{\sum}_{i=1}^{n}|\Bar{y}-y_{i}|}}, \ \Bar{y}=1/n\sum\limits_{i=1}^{n} y_{i} $$
- Determination coefficient (*R*^2^):
  17 $$ {R}^{2}=1-\text{RSE} $$

where *y* is the original signal, $\hat {y}$ is the estimated signal and *n* is the number of samples. *R*^2^ value summarises the explanatory power of the regression model.

**Fig. 10:**
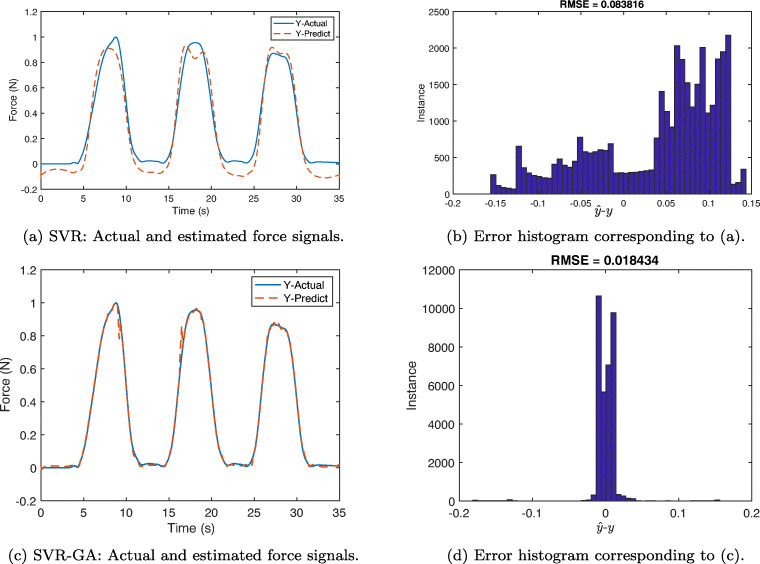
Results of applying SVR (a,b) and SVR-GA (c, d) using approach A to estimate force from sEMG signals. Solid-blue and dashed-red curves show actual and measured force data, respectively. The estimation error distribution shows out-performance of SVR-GA

In order to estimate the force signals from the measured sEMGs, we have used support vector regression. For evaluation of both SVR and SVR-GA, we have taken two different approaches. In approach A, two trials of data were collected from each subject where one is used for training the other used for testing phase. In the second approach (B), 70% of both trials from each subject were considered for training and the remaining 30% were included in the test. We performed 10-fold cross validation to randomly select these data partitions. It is noteworthy to mention that due to large number of samples in approach B, and for the sake of representation a zoomed version of force samples are given in the resulting graphs. In both experiments, we selected a Gaussian kernel with *ε* = 0.3, *C* = 0.5, and *σ*^2^ = 3 for the regression model. Figure 10 illustrates the estimated and measured force signals of S1 in addition to their corresponding RMSE using approach A in both SVR and SVR-GA methods. These results show high accuracy of the estimation process as well as small RMSE. Notably, significant improvement in reconstruction error and estimation accuracy can be observed for SVR-GA from Fig. 10(c) and (d).

**Fig. 11:**
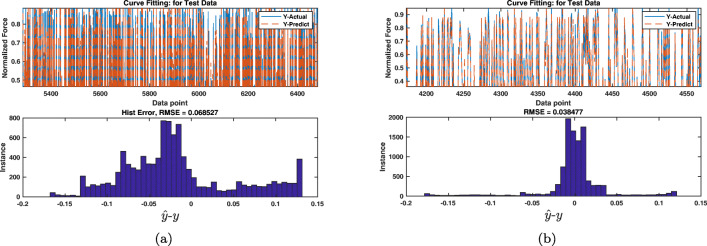
Results of applying SVR (a) and SVR-GA (b) using approach B to estimate force from sEMG signals. Solid-blue and dashed-red curves show actual and measured force data, respectively. The estimation error distribution shows out-performance of SVR-GA. Bar diagrams show the distributions of estimation error

The numerical results of conducting approaches A and B using SVR are given in Tables 5 and 6, respectively. Similarly, the associated results for SVR-GA are given in Tables 7 and 8, respectively. The performance measures in these tables have been calculated using the series of Eqs. (13), (14), (15), (16), and (17). We observe consistent results in both Tables 5 and 6 where most errors are very small and the accuracy is above 90% for most of the subjects. In addition, Table 6 shows that better *R*^2^ value is achieved using approach B (Fig. 11).

**Table 5:** SVR: different performance measures of force estimation using approach A with all subjects. The results are depicted for both train and test phases

	Train					Test				
Subject	*R*^2^ (%)	RMSE	MSE	MAE	RSE	*R*^2^ (%)	RMSE	MSE	MAE	RSE
S1	95.09	0.0806	0.0065	0.0725	0.0491	94.69	0.0838	0.0070	0.0762	0.0531
S2	94.39	0.0861	0.0074	0.0722	0.0561	93.51	0.0926	0.0086	0.0781	0.0649
S3	92.24	0.1013	0.0103	0.0912	0.0776	91.29	0.1073	0.0115	0.0994	0.0871
S4	91.16	0.1081	0.0117	0.1006	0.0884	90.73	0.1107	0.0123	0.1036	0.0927
S5	90.89	0.1089	0.0129	0.1027	0.0911	90.07	0.1147	0.0131	0.1087	0.0993

**Table 6:** SVR: different performance measures of force estimation using approach B with all subjects. The results are depicted for both train and test phases

	Train					Test				
Subject	*R*^2^ (%)	RMSE	MSE	MAE	RSE	*R*^2^ (%)	RMSE	MSE	MAE	RSE
S1	96.47	0.0684	0.0047	0.0568	0.0353	96.42	0.0685	0.0047	0.0567	0.0358
S2	95.98	0.0729	0.0053	0.0653	0.0402	95.97	0.0733	0.0054	0.0658	0.0403
S3	95.87	0.0740	0.0055	0.0663	0.0413	95.80	0.0745	0.0055	0.0669	0.0420
S4	94.75	0.0835	0.0070	0.0760	0.0525	94.64	0.0838	0.0070	0.0765	0.0536
S5	93.22	0.0947	0.0090	0.0899	0.0678	93.11	0.0953	0.0091	0.0904	0.0689

**Table 7:** SVR-GA: different performance measures of force estimation for test data using approach A with all subjects

Subject	*R*^2^ (%)	RMSE	MSE	MAE	RSE	Parameters		
						C	*ε*	*σ*^2^
S1	97.77	0.0543	0.003	0.0461	0.0223	0.034	0.013	2.4
S2	96.48	0.0682	0.0047	0.0615	0.0352	0.014	0.001	2.2
S3	96.47	0.0685	0.0047	0.0601	0.0353	0.030	0.011	2.91
S4	96.13	0.0715	0.0051	0.0632	0.0387	0.022	0.071	3.12
S5	96.10	0.0718	0.0051	0.0635	0.039	0.038	0.071	4.11

**Table 8:** SVR-GA: different performance measures of force estimation form test data using approach B with all subjects

Subject	*R*^2^ (%)	RMSE	MSE	MAE	RSE	Parameters		
						C	*ε*	*σ*^2^
S1	98.89	0.0385	0.0015	0.0209	0.0111	0.9	0.01	1.99
S2	98.85	0.0391	0.0015	0.0209	0.0209	0.9	0.1	1.6
S3	98.82	0.0814	0.0066	0.061	0.0118	0.91	0.14	1.32
S4	98.68	0.0418	0.0017	0.0237	0.0133	0.94	0.16	1.1
S5	98.15	0.1018	0.0104	0.0736	0.0185	0.94	0.11	1.86

On the other hand, Tables 7 and 8, which show the results of SVR-GA with the optimised parameters using GA, indicate higher performance compared to Tables 5 and 6. This means that automatic selection of parameters using genetic algorithm has a positive effect on the overall performance and was successful in optimising the SVR parameters. To obtain the results of these tables, we use the genetic algorithm, as explained in previous section, to optimise the parameters for each subject. Hence, we ran GA with initial population of 20 and 50 iterations. According to Tables 7 and 8, all performance measures have been significantly improved compared to those reported in Table 5 and 6.

Finally, in order to observe and compare the performance of both proposed SVR and SVR-GA methods with other relevant techniques, the average *R*^2^ value and RMSE for force estimation was calculated and is given in Table 9. In this table, the estimation metrics have been averaged over all five participants. Both methods in [50] and [51] are based on SVR but without any parameter optimisation. According to Table 9, the proposed SVR method performs slightly better than [50], whereas SVR-GA significantly outperforms both [50] and [51]. This observation supports the significance of using genetic algorithm in the proposed method to optimise the model parameters.

**Table 9:** Comparison of average *R*^2^ and RMSE obtained for the proposed method and other relevant techniques under both train and test conditions

	Train		Test	
	*R*^2^	RMSE	*R*^2^	RMSE
Proposed method (SVR)	95.25%	0.0787	95.18%	0.0791
Proposed method (SVR-GA)	98.74%	0.0604	98.67%	0.0605
Method in [50] (Gaussian kernel)	95%	6.28	94%	8.19
Method in [50] (Polynomial kernel)	92%	7.99	91%	9.82
Method in [51]	94%	–	89%	–

## Conclusions

In this paper, a knee rehabilitation robot has been designed based on the force estimation from sEMG signals. This robot composed of several parts such as electric motors and a wearable IMU sensor to measure the force proportional to the quadriceps femoris muscle at a specific knee angle. The force estimation has been studied and carried out based on the measured sEMG signals using various models, i.e. SVM, SVR, and RF. Based on the obtained results, we have observed that the model based on support vector regression with optimised parameters using genetic algorithm provide the best performance. From a physiological point of view, this non-isometric motion analysis requires describing the musculotendon length and the moment arms as a function of the joint angles. The proposed force estimation techniques increase the accuracy and performance of the therapy while muscle models become especially sensitive to the tendon stiffness and the slack length. One limitation of the proposed study is small number of subjects to collect the data. This may limit the applicability of some of the learning-based methods, e.g. deep neural networks, which requires large-scale datasets. Another limitation of the current system is off-line EMG data collection. For future work, we constantly recruit more volunteers to augment our database. Also, we are planning to optimise the coding to be able to implement online force estimation and EMG data collection instead of using prerecorded sEMG signals.

## Footnotes

1 http://www.seniam.org
